# Metabolites extracted from microorganisms as potential inhibitors of glycosidases (α-glucosidase and α-amylase): A review

**DOI:** 10.3389/fmicb.2022.1050869

**Published:** 2022-11-17

**Authors:** Xiaojing Wang, Jiaying Li, Jiaqi Shang, Jing Bai, Kai Wu, Jing Liu, Zhijun Yang, Hao Ou, Lei Shao

**Affiliations:** ^1^Affiliated Zhoupu Hospital, Shanghai University of Medicine and Health Sciences, Shanghai, China; ^2^Microbial Pharmacology Laboratory, Shanghai University of Medicine and Health Sciences, Shanghai, China; ^3^School of Pharmacy, Shanghai University of Medicine and Health Sciences, Shanghai, China; ^4^Shanghai University of Medicine and Health Science, Shanghai University of Traditional Chinese Medicine, Shanghai, China; ^5^School of Medical Instrument and Food Engineering, University of Shanghai for Science and Technology, Shanghai, China; ^6^School of Chemistry and Life Sciences, Suzhou University of Science and Technology, Suzhou, China; ^7^School of Medical Technology, Shanghai University of Medicine and Health Sciences, Shanghai, China; ^8^Department of Critical Care Medicine, The Third Xiangya Hospital, Central South University, Changsha, Hunan, China

**Keywords:** α-glucosidase inhibitor, α-amylase inhibitor, microbes, endophytes, marine microorganism, medical application

## Abstract

α-Glucosidase and α-amylase are the two main glycosidases that participate in the metabolism of carbohydrates. Inhibitors of these two enzymes are considered an important medical treatment for carbohydrate uptake disorders, such as diabetes and obesity. Microbes are an important source of constituents that have the potential to inhibit glycosidases and can be used as sources of new drugs and dietary supplements. For example, the α-glucosidase inhibitor acarbose, isolated from *Actinoplanes* sp., has played an important role in adequately controlling type 2 diabetes, but this class of marketed drugs has many drawbacks, such as poor compliance with treatment and expense. This demonstrates the need for new microorganism-derived resources, as well as novel classes of drugs with better compliance, socioeconomic benefits, and safety. This review introduces the literature on microbial sources of α-glucosidase and α-amylase inhibitors, with a focus on endophytes and marine microorganisms, over the most recent 5 years. This paper also reviews the application of glycosidase inhibitors as drugs and dietary supplements. These studies will contribute to the future development of new microorganism-derived glycosidase inhibitors.

## Introduction

Enzyme inhibitors have attracted increasing attention for medical applications. The common targets of such inhibitors are glycosidases, β-lactamases, lipases, proteases, and xanthine oxidase (Usman et al., 2019). Glycosidases (EC 3.2.1) contain α-glucosidase (EC 3.2.1.20) and α-amylase (EC 3.2.1.1), which are mainly involved in carbohydrate metabolism (Aslan et al., 2018; Demir et al., 2018). α-Glucosidases hydrolyze terminal non-reducing residues of various carbohydrate substrates, resulting in the release of α-glucopyranose (Baron, 1998; Heacock et al., 2005). Meanwhile, α-amylases hydrolyze α-D-(1,4)-glucan linkages in starch to form polymers composed of glucose units (Paul et al., 2021). Since glycosidases are widely used to hydrolyze carbohydrates, inhibitors of these two enzymes are beneficial to treatment carbohydrate-dependent diseases like diabetes, obesity and other diseases (Asano, 2003; Usman et al., 2019). Some glycosidase inhibitors are also considered potential useful tools for the treatment of viral infections and lysosomal-storage-diseases (Block et al., 1998; Chang et al., 2013).

Glycosidase inhibitors hitherto have mostly been derived from microbial sources, which can be easily handled on a large scale, as compared to that with plants (Shah et al., 2018). Since the classic α-glucosidase inhibitor nojirimycin, derived from microbial resources, was first reported by Niwa et al. (1970), more and more glycosidase inhibitors have been isolated from microorganisms (Niwa et al., 1970). However, the search for novel microorganisms with new glycosidase inhibitors was previously concentrated on general environment, which has led to microbial resource scarcity (Bisaria et al., 2020). Therefore, the discovery of new inhibitors from uncommon environments, such as the marine ecosystem and endophytic microorganisms, has been the focus in recent years (Davies-Bolorunduro et al., 2021; Karthikeyan et al., 2022).

The marine environment has not been fully explored, and metabolites of marine microbial origins have a more important role in drug discovery (Pathak et al., 2022). These natural products from marine microorganisms have various bioactivities, such as anti-bacterial (Yang et al., 2018), anti-fungal (Sangkanu et al., 2021), anti-viral (Richard et al., 2018), anti-parasitic (Santos et al., 2019), anti-tumor (Yan et al., 2020), and anti-diabetic properties (Elaiyaraja et al., 2018). The survival of bacteria that live in unusual marine ecosystem depends on these active metabolites (Davies-Bolorunduro et al., 2021).

Endophytic microorganisms, which have symbiotic relationships with plants without causing harm, are a prospective source of novel drugs with various bioactivities (Manganyi et al., 2019). Through their interactions with plants, endophytes generate several metabolites with pharmaceutical applications, such as anti-bacterial (Deshmukh et al., 2022), anti-fungal (Deshmukh et al., 2018), anti-viral (Lacerda et al., 2022), anti-cancer (Gangadevi and Muthumary, 2009; Gill and Vasundhara, 2019), anti-oxidant (Toghueo and Boyom, 2019), and anti-diabetic compounds (Agrawal et al., 2022). The utilization of these endophytes for discovering new active metabolites has been increasing, and it ultimately will be of major importance for pharmaceutical, medical, and industrial applications (Lopéz et al., 2019; Qiu et al., 2019).

The aim of the present review was to consolidate the literature on microbial sources of α-glucosidase and α-amylase inhibitors from 2018 to 2022. Peer-reviewed articles were retrieved from searches using the following databases: PubMed, Google Scholar, and Web of Science.

## Inhibitors of glycosidases from different microbes

### Inhibitors of glycosidases from bacteria

Bacteria are the main traditional sources of glycosidase inhibitors and bacteria with inhibitory activity against glycosidase in recent 5 years are listed in Table 1.

**Table 1:** Bacteria with inhibitory activity against glycosidase in recent 5  years.

Microbial resources	Production identified	Type	Reference
Actinomycetes			
*Streptomyces*			
*S. costaricanus* EBL.HB6	α-glucosidase inhibitor	–	Nguyen et al. (2021)
*S. coelicoflavus* SRBVIT13	α-glucosidase inhibitor (compound)	marine	Kumar and Rao (2018)
*Streptomyces* sp.S2A	α-glucosidase inhibitor and α-amylase inhibitor (compound)	marine	Siddharth and Vittal (2018)
*Streptomyces* sp.SCA29	α-glucosidase inhibitor and α-amylase inhibitor inhibitor(compound)	marine	Siddharth and Vittal (2019)
*S. koyangensis* strain B025	α-amylase inhibitor	endophytic	Saini and Gangwar (2018)
*Streptomyces* sp. SCSIO 40064	α-glucosidase inhibitor (compound)	marine and endophytic	Chen et al. (2022)
*Streptomyces* strain	α-glucosidase inhibitor and α-amylase inhibitor	marine and endophytic	El-Gendy et al. (2022)
*Streptomyces* strain	α-amylase inhibitor	endophytic	Dat and Thuy (2021)
*Streptomyces* sp. TD-X10	α-glucosidase inhibitor and α-amylase inhibitor	–	Chi et al. (2020)
*Streptomyces* sp. TD-X13	α-glucosidase inhibitor	–	Chi et al. (2020)
*Amycolatopsis thermoflava* strain SFMA-103	α-glucosidase inhibitor and α-amylase inhibitor (compound)	–	Chandrasekhar et al. (2021)
*Nocardiopsis* SCA21	α-glucosidase inhibitor and α-amylase inhibitor (compound)	marine	Siddharth and Rai (2019)
*Arthrobacter enclensis*	α-glucosidase inhibitor (compound)	marine	Mawlankar et al. (2020)
*Saccharomonospora* oceani VJDS-3	α-amylase inhibitor and α-glucosidase inhibitor (compound)	endophytic	Indupalli et al. (2018)
*Actinomycete*	α-glucosidase inhibitor and α-amylase inhibitor	endophytic	(Saini and Gangwar)
*Bacillus*			
*Bacillus* strain	α-amylase inhibitor	endophytic	Dat and Thuy (2021)
*Bacillus* sp. RAR_M1_45	α-glucosidase inhibitor and α-amylase inhibitor	endophytic	Dat et al. (2022)
*Bacillus* sp. TD-V21; *Bacillus* sp. TD-V24	α-glucosidase inhibitor	–	Chi et al. (2020)
*Lactobacillus*			
*Lactobacillus pentosus* TL 2.7, TL 5.8 and TL 7.8	α-glucosidase inhibitor and α-amylase inhibitor	endophytic	Frediansyah et al. (2019)
*Lactobacillus sakei* Probio65; *Lactobacillus plantarum* Probio-093	α-glucosidase inhibitor and α-amylase inhibitor	–	Gulnaz et al. (2021)
*Others*			
*Enterobacter cloacae TD-V20*	α-glucosidase inhibitor	–	Chi et al. (2020)
Gram-negative bacteria	α-amylase inhibitor	endophytic	Pujiyanto et al. (2018)
*Pseudovibrio* strain	α-amylase inhibitor	endophytic	Dat and Thuy (2021)

Compound: the researchers isolated the compound from strains.

#### Actinomycetes

In general, secondary metabolites obtained from bacteria exhibit various activities (Solecka et al., 2012). Actinomycetes, especially *Streptomyces* species, such as *Streptomyces hygroscopicus* (validamycin; Kameda et al., 1984), *Streptomyces lavendulae* (1-deoxynojirimycin, DNJ; Murao and Miyata, 1980; Ezure et al., 1985), and *Streptomyces dimorphogenes* (trestatin; Yokose et al., 1983), are well known to produce novel glycosidase inhibitors of therapeutic value.

##### Streptomyces

In the most recent 5 years, most glycosidase inhibitors were still produced by *Streptomyces* species, and these are very potent. α-Glucosidase inhibitors from *Streptomyces costaricanus* EBL.HB6 were isolated and purified by Nguyen et al. (2021) in Vietnam. The IC_50_ value of purified inhibitors was 9.59 mg/ml. They also optimized cultivation conditions to increase the yield of inhibitors. However, very few *Streptomyces* strains with glycosidase inhibitory activity have originated from the general environment in recent years, and most of them are derived from the marine ecosystem and endophytic microorganisms.

**Figure 1:**
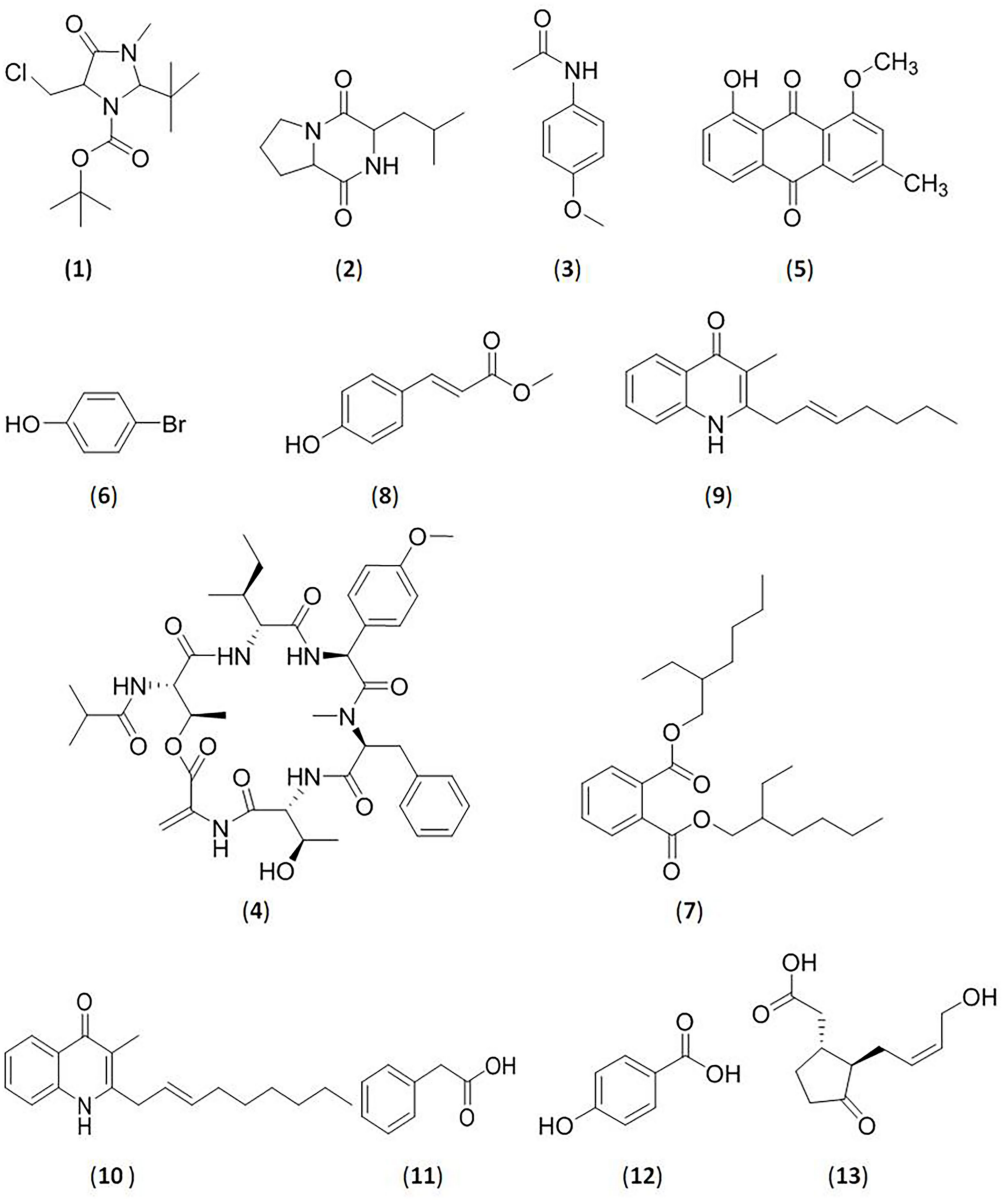
Glycosidase inhibitors compounds from bacteria. (**1**).2-t-butyl-5-chloromethyl-3-methyl-4-oxoimidazolidine-1-carboxylic acid, t-butyl ester; (**2**).pyrrolo[1–a]pyrazine-1,4-dione,hexahydro-3-(2-methylpropyl); (**3**).4-methoxyacetanilide; (**4**).isobutyryl hexapeptide TXS-2; (**5**).1-O-methyl chrysophanol (OMC); (**6**).bromophenol derivative; (**7**).bis (2-ethylhexyl) phthalate; (**8**).methoxy ethyl cinnamate (ethyl (E)-3-(4-methoxyphenyl)acrylate; (**9**)2-(2-heptenyl)-3-methyl-4-quinolinone; (**10**).3-methyl-2-(2-nonenyl)-4-quinolinone; (**11**).2-phenylacetic acid; (**12**).4-hydroxybenzoic acid; (**13**). (−)-jasmonic acid.

The deep-ocean interior contains many new *Streptomyces* strains, which show strong inhibitory activity against glycosidases. Kumar and Rao (2018) confirmed that the extract of the marine organism *Streptomyces coelicoflavus* SRBVIT13 exhibits remarkable inhibitory activity against α-glucosidase of yeast and mammals *in vitro*. *In vivo*, postprandial blood glucose levels decreased after the oral administration of this extract in diabetic rats. The main compound with activity was identified as 2-t-butyl-5-chloromethyl-3-methyl-4-oxoimidazolidine-1-carboxylic acid, t-butyl ester (**1**). Marine-derived *Streptomyces* sp.S2A was isolated from the gulf of India by Siddharth and Vittal. The active metabolites were extracted, and the α-glucosidase and α-amylase inhibitory activities were tested, with IC_50_ values of 21.17 μg/ml and 20.46 μg/ml, respectively. The bioactive compound was determined to be pyrrolo[1–a]pyrazine-1,4-dione,hexahydro-3-(2-methylpropyl) (**2**) (Siddharth and Vittal, 2018). Siddharth and Vittal (2019) further extracted a bioactive fraction from marine *Streptomyces* sp.SCA29 in India. The fraction was assayed for inhibitory activity against α-glucosidase and α-amylase, and the IC_50_ values were 44.26 μg/ml and 53.19 μg/ml, respectively. 4-Methoxyacetanilide (**3**), an acetamide derivative, was also purified based on a bioassay-guided method (Figure 1).

Endophytes from *Murraya koenigii* comprise a library of novel *Streptomyces* strains. *M. koenigii* is a small, deciduous shrub that possesses notable pharmacological effects, such as anti-diabetic, anti-microbial, and anti-diarrheal (Tembhurne and Sakarkar, 2010). Saini confirmed that the endophytic *Streptomyces koyangensis* strain B025 isolated from *M. koenigii* has remarkable inhibitory activity against α-amylase. The active compounds were considered to be phenols (Saini and Gangwar, 2018).

For several years, marine endophytic *Streptomyces* strains isolated from different marine sources comprise one of the most popular research subjects (Chen et al., 2016). The isobutylhexapeptide TXS-2(**4**) was isolated from the marine *Streptomycetes* SCSIO 40064 by Chen et al. (2022). They found that this compound significantly inhibited the activity of α-glucosidase, and the IC_50_ value was 18.67 ± 1.27 mM. El-Gendy et al. (2022) demonstrated the effects of a new endophytic *Streptomyces* species from the marine *Sarcophyton convolutum*. The strain displayed α-glucosidase inhibitory activity with an IC_50_ value ≥ 84.34 ± 2.25% and α-amylase inhibitory activity ≥ 88.20 ± 1.33%.

##### Non-*Streptomyces*-actinomycetes

In addition to *Streptomyces* species, glycosidases inhibitors can also be derived from non-*Streptomyces*-actinomycetes, such as *Actinoplanes utahensis* (acarbose; Schwientek et al., 2012) and others. In recent years, researchers have isolated inhibitors from *Amycolatopsis*, *Nocardiopsis*, *Arthrobacter*, *Saccharomonospora*, and other strains in succession. Most of them have come from marine or plant endophytic microorganisms, which are similar to *Streptomyces* species.

An active compound was isolated from *Amycolatopsis thermoflava* SFMA-103 by Chandrasekhar et al. (2021). The compound was identified as 1-O-methyl chrysophanol (OMC, **5**), a class of hydroxyanthraquinones. OMC inhibited carbohydrate metabolizing enzymes *in vitro*, with IC_50_ values of 38.49 μg/ml (α-glucosidase) and 3.4 mg/ml (α-amylase). When orally administered Wistar rats, OMC was demonstrated to inhibit the increase of blood sugar levels in vivo.

*Nocardiopsis* SCA21 was isolated from marine sediment in India by Siddharth and Rai (2019). Two bioactive compounds were first purified and identified as 4-bromophenol, a bromophenol derivative (**6**), and Bis (2-ethylhexyl) phthalate, a phthalate ester (**7**). These two compounds exhibited strong enzyme inhibitory activities against α-glucosidase, with IC_50_ values of 94.61 μg/ml (compound **6**) and 202.33 μg/ml (compound **7**). Compound **6** could also could inhibit the activity of α-amylase with an IC_50_ value of 103.23 μg/ml, whereas compound **7** was less active against α-amylase with an IC_50_ value >250 μg/ml. Mawlankar et al. (2020) reported a bioactive substance from the novel marine species *Arthrobacter enclensis*. The purified compound was found to be a α-glucosidase inhibitor, and its IC_50_ value was 500 ± 0.142 μg/ml. It was identified as a C7N aminocyclitol, that displayed high similarity to acarbose, but the structure has not been completely solved. Researchers have also demonstrated that its encoding gene is similar to the biosynthetic gene cluster encoding acarbose (7%).

Indupalli et al. (2018) first reported an unusual actinobacterium *Saccharomonas* oceani VJDS-3 isolated from the mangrove forest in India. The ethyl methoxycinnamate (ethyl (E)-3-4-methoxyphenyl) acrylate (**8**) was purified. It exhibited significant inhibitory activity against α-glucosidase at 20 μg/ml with an IC_50_ value of 66.8 ± 1.2 μg/ml and moderate to weak inhibitory activity against α-amylase at 40 μg/ml with an IC_50_ value of 11.5 ± 0.5 μg/ml. Saini and Gangwar (2022) obtained an endophytic actinomycete strain derived from *Aegle marmelos*. The ethyl acetate extract was assayed to have IC_50_ values of 1950.71 ± 0.11 μg/ml against α-amylase, and 391.38 ± 0.09 μg/ml against α-glucosidase. The results indicated that the crude extract has the potential to reduce postprandial blood glucose. However, further studies are needed to identify the strain and the active substance.

#### Other bacteria

In addition to actinomycetes, other bacteria were reported to produce glycosidase inhibitors, such as *Bacillus* sp. (nojirimycin; Iida et al., 1987), *Vibrio* sp. (El-Hady et al., 2017), and lactic acid bacteria (Nurhayati et al., 2017). Scientists have discovered many glycosidase inhibitors from *Bacillus* sp.*, Lactobacillus* sp., and other bacteria in the past 5 years.

*Bacillus* species can produce secondary metabolites with various activities, which enable the bacterium to resist external harmful factors (Sansinenea and Ortiz, 2011; Yang et al., 2014). Five endophytes from *Rhizophora stylosa* roots with high α-amylase inhibitory activity were reported by Dat and Thuy. They were identified as *Bacillus*, *Streptomyces* and *Pseudomvibrio*. The ethyl acetate extracts of five isolated strains showed inhibitory activities against α-amylase with values ranging from 31.4 ± 3.1% to 59.7 ± 6.4% (Dat and Thuy, 2021). Further, Dat et al. (2022) investigated an endophytic *Bacillus* sp. RAR_M1_45 derived from the mangrove plant *Rhizophora apiculata Blume*. They isolated five compounds from the crude extract of *Bacillus* sp., that is 2-(2-heptenyl)-3-methyl-4-quinolinone (**9**), 3-methyl-2-(2-nonenyl)-4-quinolinone (**10**), 2-phenylacetic acid (**11**), 4-hydroxybenzoic acid (**12**), and (−)-jasmonic acid (**13**). All five compounds showed inhibitory activity against α-glucosidase with IC_50_ values ranging from 163.3 ± 10.66 μg/ml to 960.4 ± 8.62 μg/ml and inhibitory activity against α-amylase with IC_50_ values ranging from 63.87 ± 4.23 μg/ml to 649.9 ± 17.5 μg/ml.

*Lactobacillus* sp. is another important source of glycosidase inhibitors. Frediansyah et al. (2019) found that all three cell extracts and cell-free supernatants of *Lactobacillus pentosus* strains isolated from *Muntingia calabura* L. have inhibitory activity against α-glucosidase and α-amylase *in vitro*. Moreover, compared to that in the cell-free supernatant group, the cell extracts exhibited higher inhibition of α-glucosidase. However, it seemed to have the opposite effect on the inhibition of α-amylase. In addition, Gulnaz et al. (2021) reported that an extract of *Lactobacillus sakei* Probio65 and *Lactobacillus plantarum* Probio-093 has inhibitory activity against α-glucosidase and α-amylase. These two *Lactobacillus* strains could change the intestinal microbiota diversity in high-fat diet-induced diabetic mice, and Probio-093 had a more significant effect on the intestinal microbiota. Together, this showed that *Lactobacillus* comprises potential candidates to treat type 2 diabetes.

Chi et al. (2020) screened five microbial strains in Vietnam with glycosidase inhibitory effects. The strains were identified as and named *Enterobacter cloacae* TD-V20, *Bacillus* sp. TD-V21, *Bacillus* sp. TD-V24, *Streptomyces* sp. TD-X13, and *Streptomyces* sp. TD-X10. All extracts of the five strains exhibited inhibitory activities against α-glucosidase, and *Streptomyces* sp. TD-X10 showed stronger inhibitory activity against α-amylase. Eleven endophytic bacterial strains from *Annona muricata* were found to inhibit α-amylase activity. One strain (DS21) exhibited the highest activity with a 72.22% inhibition rate. The strain was a gram-negative bacterium, and needed to be further identified (Pujiyanto et al., 2018).

### Inhibitors of glycosidases from fungus

Saito reported an α-amylase inhibitor produced by the fungus *Cladosporium herbarum* F-28, in contrast to the traditional opinions that only bacteria can produce glycosidase inhibitors, and this inhibitor was found to have high specificity for mammalian amylase (Saito, 1982). Further, glycosidase inhibitory activities were tested using *Penicillium* (Kwon et al., 2000), the endophytic fungus *Stemphylium globuliferum* from *Trigonella foenum-graceum* (Pavithra et al., 2014), *Aspergillus awamori* isolated from *Acacia nilotica* (proteinaceous α-glycosidase inhibitor; Singh and Kaur, 2016; Singh et al., 2021), and other fungi. Compared to bacteria, more fungal resources have been found in the past 5 years, including *Aspergillus, Penicillium, Mycosphaerella, Alternaria* and mushrooms (macrofungi), which make many novel compounds with high inhibitory activity against glycosidases (Table 2).

**Table 2:** Fungus with inhibitory activity against glycosidase in recent 5  years.

Microbial resources	Production identified	Type	Reference
*Aspergillus*			
*Aspergillus terreus* OUCMDZ-2739	α-glucosidase inhibitor (new compound)	marine	Sun et al. (2018)
*Aspergillus egypticus* HT166S	α-amylase inhibitor (compound)	endophytic	Ruzieva et al. (2020)
*Aspergillus* sp. (MAN)	α-amylase inhibitor	endophytic	Khan et al. (2019)
*Penicillium*			
*Penicillium* sp. TW58-16	α-glucosidase inhibitor (new compound)	marine	Gou et al. (2021)
*Penicillium canescens*	α-glucosidase inhibitor (new compound)	endophytic	Malik et al. (2020)
*Penicillium pinophilum* SCAU037	α-glucosidase inhibitor (compound)	endophytic	He et al. (2019)
*Penicillium* TR3	α-glucosidase inhibitor and α-amylase inhibitor (compound)	endophytic	Siregar et al. (2022)
*Mycosphaerellaceae*			
*Mycosphaerella* sp. SYSU-DZG01	α-glucosidase inhibitor (new compound)	endophytic	Qiu et al. (2019)
*Mycosphaerellaceae Zasmidium* sp. EM5-10	α-glucosidase inhibitor	endophytic	Lopéz et al. (2019)
*Alternaria*			
*Alternaria* sp. QPS 05	α-glucosidase inhibitor (compound)	endophytic	Indrianingsih et al. (2018)
*Alternaria* sp. (JCO)	α-amylase inhibitor	endophytic	Khan et al. (2019)
*Mushroom*			
*Grifola frondosa*	α-glucosidase inhibitor (compound)	–	Chen et al. (2018)
oyster mushroom	α-amylase inhibitor (compound)	–	Tamboli et al. (2018)
*Dacryopinax spathularia* and *Schizophyllum commune*	α-amylase inhibitor	–	Kumar et al. (2018)
*Inonotus obliquus* (Ach. ex Pers.) Pilát	α-amylase inhibitor	–	Stojkovic et al. (2019)
*Others*			
*Nigrospora sphaerica*	α-glucosidase inhibitor (new compound)	endophytic	Ukwatta et al. (2019)
*Schizophyllum commune* Fr.	α-glucosidase inhibitor (compound)	endophytic	Sharma et al. (2021)
*Diaporthe eres* (SPEF004)	α-glucosidase inhibitor and α-amylase inhibitor	endophytic	Saravanakumar et al. (2021)
*Colletotrichum*	α-glucosidase inhibitor and α-amylase inhibitor	endophytic	Roopa et al. (2022)
*Talaromyces indigoticus* FS688	α-glucosidase inhibitor (compound)	marine	Li et al. (2021)
*Fuscoporia torulosa* MFSLP-12	α-glucosidase inhibitor and α-amylase inhibitor	–	Alcantara et al. (2019)
MBR, NGU and NTH	α-amylase inhibitor	endophytic	Khan et al. (2019)

Compound: the researchers isolated compounds from strains. New compound: the researchers isolated new compounds from strains.

#### Aspergillus

**Figure 2:**
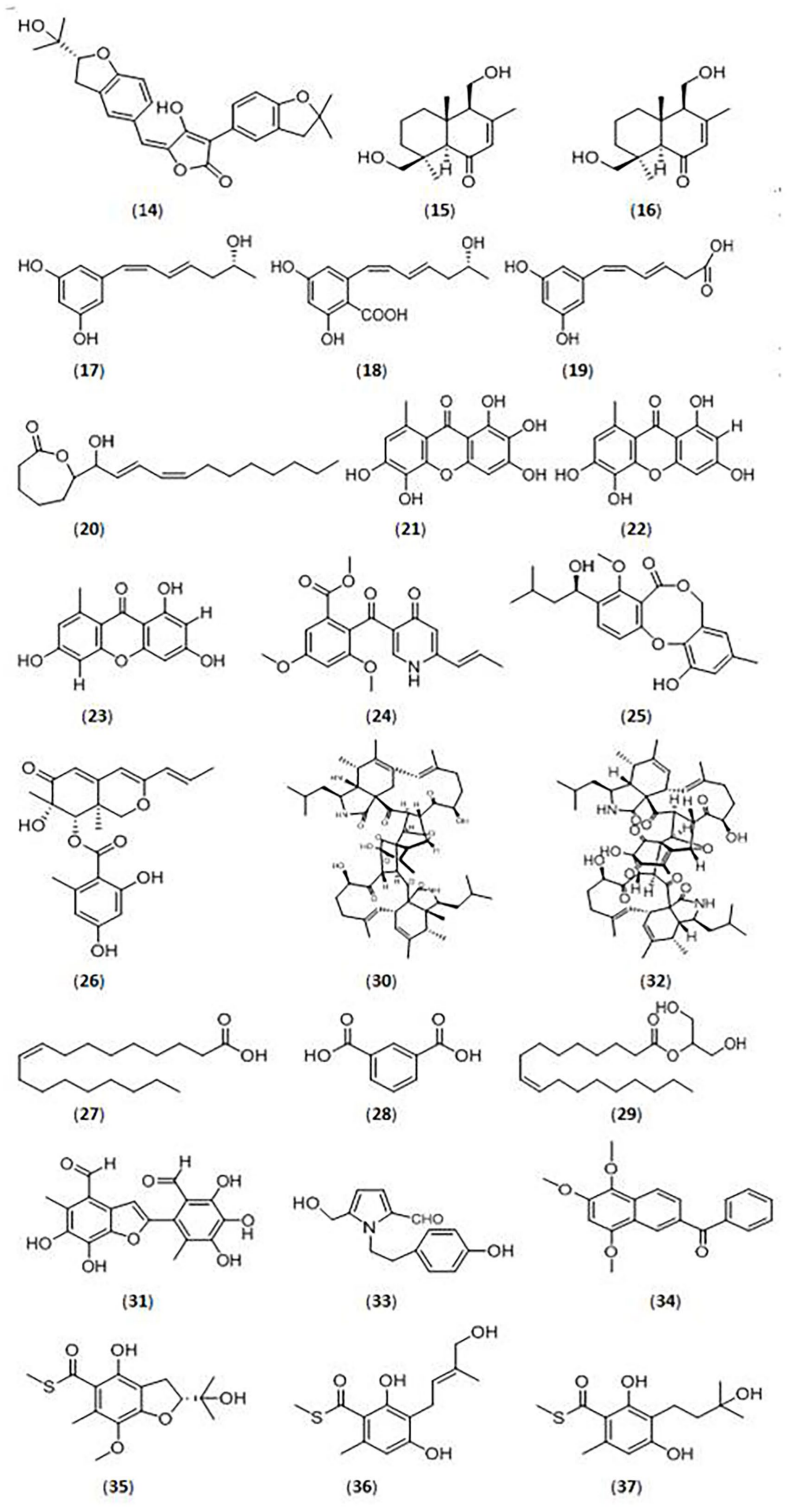
Glycosidase inhibitors compound from fungi. (**14**).(R,E)-3-(2,2-dimethyl chroman6-yl)-4-hydroxy-5-((2-(2-hydroxypropan-2-yl)-2,3-dihydrobenzofuran-5-yl)methylene) furan-2(5H)-one; (**15**).(4S,5R,9S,10R)-11,13-dihydroxy-drim-7-en-6-one; (**16**).5-((R,1Z,3E)-6-hydroxy-1,3-heptadien-1-yl)-1,3-benzenediol; (**17**).4-carboxy-5-((R,1Z,3E)-6-hydroxy-1,3-heptadien-1-yl)- 1,3-benzenediol; (**18**).4-carboxy-5-((1Z,3E)-1,3-heptadien1-yl)-1,3-benzenediol; (**19**). (1Z,3E)-4-carboxy-1,3-butadienyl-1-yl)-1,3-benzenediol; (**20**).ε-caprolactone derivative; (**21**).unreported xanthone; (**22–23**).xanthones; (**24**).vermistatin; (**25**).penicillide; (**26**).Sch725680; (**27**).oleic acid; (**28**).1,3-benzenedicarboxylic acid; (**29**).hexadecanoic acid; (**30**).asperchalasine I; (**31**).epicoccolide B; (**32**).asperchalasine A; (**33**).5-hydroxymethyl-1-[2-(4-hydroxyphenyl)-ethyl]-1H-pyrrole-2-carbaldehyde; (**34**).nigronapthaphenyl; (**35**).eurothiocins C; (**36**).eurothiocins F; (**37**).eurothiocins G.

*Aspergillus* species are a source of bioactive secondary metabolites, especially marine-derived and endophytic strains. Sun et al. (2018) identified new meroterpenoids (R,E)-3-(2,2-dimethyl chroman6-yl)-4-hydroxy-5-((2-(2-hydroxypropan-2-yl)-2,3-dihydrobenzofuran-5-yl)methylene) furan-2(5H)-one (**14**) isolated from the marine fungus *Aspergillus terreus* OUCMDZ-2739, which showed significant inhibitory activity against α-glucosidase with an IC_50_ value of 24.8 μM. They also tested the known compounds rubrolide S and butyrolactone I from *A. terreus,* which also exhibited stronger α-glucosidase inhibitory activity, with IC_50_ values of 1.2 μM and 61.6 μM, respectively (Figure 2).

The *Aspergillus egypticus* HT166S (endophytic fungus) was isolated from the plant *Helianthus tuberosus* by Ruzieva et al. (2020). Methanol extracts were found to possess α-amylase inhibitory activity with 75.4% inhibition rate. The methanol component contained flavonoids, terpenoids, anthraquinones, tannins and other bioactive compounds, and flavonoids had the highest activity.

#### Penicillium

*Penicillium* species are traditional glycosidase inhibitor-producing bacteria. Gou et al. (2021) identified 18 compounds (eight new and ten known compounds) from a marine fungus *Penicillium* sp. TW58-16. Among them, five new compounds ((4S,5R,9S,10R)-11,13-dihydroxy-drim-7-en-6-one (**15**), 5-((R,1Z,3E)-6-hydroxy-1,3-heptadien-1-yl)-1,3-benzenediol (**16**), 4-carboxy-5-((R,1Z,3E)-6-hydroxy-1,3-heptadien-1-yl)- 1,3-benzenediol(**17**), 4-carboxy-5-((1Z,3E)-1,3-heptadien1-yl)-1,3-benzenediol(**18**), and 5-((1Z,3E)-4-carboxy-1,3-butadienyl-1-yl)-1,3-benzenediol (**19**)) showed significant α-glucosidase inhibitory activities, with inhibition rates from 32.0 to 74.4%, and a ε-caprolactone derivative (**20**) showed the highest inhibitory effects (91.1%).

Malik et al. (2020) investigated an endophytic *Penicillium canescens* strain isolated from *Juniperus polycarpos* fruits, and identified three xanthones (one novel xanthone (**21**) and another two known xanthones (**22–23**)) with inhibitory effects against α-glucosidase. The three compounds displayed α-glucosidase inhibitory activities, with IC_50_ values from 32.32 ± 1.01 μM to 75.20 ± 1.02 μM.

The endophytic fungus *Penicillium pinophilum* SCAU037, isolated from mangrove, was studied by He et al. (2019). Three compounds, vermistatin (**24**), penicillide (**25**), and Sch725680 (**26**), were purified and showed stronger α-glucosidase inhibitory activity with IC_50_ values of 51.9 μM, 78.4 μM, and 33.8 μM, respectively.

Siregar et al. isolated four endophytic fungi (*Penicillium* and *Aspergillus*) from the Raru plant (Situmorang et al., 2015). The *Penicillium* TR3 strain displayed significant α-amylase inhibitory activity with an inhibition rate of 96.34%. They separated and identified several compounds, and compound **27** (oleic acid; Ahmad et al., 2012; Indrianingsih et al., 2018), compound **28** (1,3-benzenedicarboxylic acid, bis (2-ethylhexyl) ester, isophthalic acid group; Rajan and Aanandhi, 2017; Abusufyan et al., 2018), compound **29** (hexadecanoic acid, 2-hydroxy-1-(hydroxymethyl) ethyl ester (CAS), palmitin group; Venkatramanan et al., 2020) showed inhibitory activities against α-glucosidase and α-amylase (Siregar et al., 2022).

#### Mycosphaerellaceae

Researchers have reported endophytic Mycosphaerellaceae strains as a source of glycosidase inhibitors, and have isolated novel compounds from them in the most recent 5 years. Qiu et al. (2019) isolated three compounds (asperchalasine I(**30**), epicoccolide B(**31**), asperchalasine A(**32**)) from the mangrove fungus *Mycosphaerella* sp. SYSU-DZG01, which showed stronger α-glucosidase inhibitory activity with IC_50_ values 17.1, 26.7, and 15.7 μM, respectively. Moreover, asperchalasine I (**30**) was a novel compound with significant α-glucosidase inhibitory activity, indicating its medicinal potential.

Lopéz et al. (2019) studied the endophytic Mycosphaerellaceae fungus *Zasmidium* sp. EM5-10 isolated from mangrove leaves. The crude extract of this fungus showed stronger α-glucosidase inhibitory activity, with an inhibition rate of 91.3%.

#### Alternaria

Endophytic *Alternaria* species were also found to be active against different glycosidases. The endophytic fungus *Alternaria* sp. QPS 05, with strong inhibitory activity against α-glucosidase, was isolated from *Quercus phillyraeoides* A. Gray by Indrianingsih et al. Researchers separated a fatty acid extract with significant α-glucosidase inhibitory activity. Further studies revealed that the mixture contained linoleic acid, oleic acid, palmitic acid, and linolenic acid (Indrianingsih et al., 2018).

Khan et al. (2019) reported an aqueous extract of the endophytic fungus JCO (*Alternaria* sp.), isolated from *Syzygium cumini* L (jambolana), which had stronger inhibitory activity against α-amylase (62%). Further, the endophytic fungi MAN (*Aspergillus* sp.), MBR, isolated from *Mangifera indica* (mango), and NGU, NTH, isolated from *Azadirachta indica* (neem) exhibited α-amylase inhibitory activity from 49 to 59%.

#### Mushrooms

Mushrooms are fungi which have broad application as functional foods and medicinal products based on their active compounds (Papoutsis et al., 2021). Many scientists have demonstrated that different mushrooms have strong anti-diabetic, antioxidant, anti-viral, anti-tumor, and immunoregulatory bioactivities (Thakur, 2020). Chen et al. isolated many compounds from *Grifola frondosa*, a type of wild mushroom. Further studies demonstrated that compound 5-hydroxymethyl-1-[2-(4-hydroxyphenyl)-ethyl]-1H-pyrrole-2-carbaldehyde (**33**) showed significant inhibitory activities against α-glucosidase from baker’s yeast and mammalian intestines, with IC_50_ values of 44.42 μM ± 5.11 μM and 28.65 μM ± 3.14 μM, respectively (Chen et al., 2018; Papoutsis et al., 2021).

An edible oyster mushroom was also collected from India, and its inhibitory activity against α-amylase was studied by Tamboli et al. (2018). Different extracts were prepared, and methanol, acetone, and chloroform extracts showed inhibitory activities against α-amylase, with IC_50_ values of 383, 224, and 1.71 μg/ml, respectively. Further studies on its active composition showed that flavonoids in the acetone extract and glycoproteins in the chloroform extract could be the active components.

Kumar et al. (2018) tested extracts from two edible macrofungi (*Dacryopinax spathularia* and *Schizophyllumcommune*), for their inhibitory activities against α-amylase. The extract of *D. spathularia* showed an α-amylase inhibition rate of 38.24% at 1000 μg/ml, and the extract of *S. commune* had a rate of 48.19%.

Stojkovic et al. (2019) investigated the *in vitro* anti-diabetic properties of six edible and medicinal mushroom species, *Agaricus blazei Murrill*, *Coprinus comatus*, *Cordyceps militaris*, *Inonotus obliquus*, *Morchella conica* and *Phellinus linteus*. The methanol extract of *C. comatus* showed the highest inhibitory activity against α-amylase with an IC_50_ value of 714.45 μg/ml. All of the six tested macrofungi had inhibitory effects against α-glucosidase, and the strongest inhibitory effect was found with *I. obliquus*, with an IC_50_ value of 220.31 μg/ml, which was the most potent strain.

#### Other fungi

A variety of other fungi, especially endophytic fungi, were also discovered for their inhibitory activities against glycosidases in recent years. Nigronapthaphenyl (**34**), a new compound extracted from the endophytic fungus *Nigrospora sphaerica* derived from the mangrove *Bruguiera gymnorrhyza* was reported by Ukwatta et al. (2019). The new substance displayed strong inhibitory activity against α-glucosidase (IC_50_ value of 6.9 ± 0.5 μM). Sharma et al. (2021) isolated the endophytic fungus *Schizophyllum commune* Fr. from *Aloe vera* and its extract showed more than 90% inhibitory activity against α-glucosidase. Treatment of STZ-induced diabetic rats with the fungus extract reduced blood glucose levels. Phenols and terpenoids were identified in the ethyl acetate extract, which could be the active ingredients. Saravanakumar et al. (2021) surveyed the glycosidase inhibitory activities of the endophyte *Diaporthe eres* (SPEF004) derived from the *Ligustrum obtusifolium* leaf. The ethyl acetate extract of *D. eres* displayed α-glucosidase inhibitory activity of 13.28 ± 0.94% and α-amylase inhibitory activity of 41.11 ± 1.52%. An endophytic fungal *Colletotrichum* species derived from *Salacia macrosperma* was detected by Roopa et al. (2022). The fungal extract showed inhibitory effects against α-glucosidase and α-amylase with IC_50_ values of 124.62 and 106.11 μg/ml, respectively.

The marine fungus *Talaromyces indigoticus* FS688 was studied by Li et al. (2021), and many bioactive compounds were isolated. The compound eurothiocins C (**35**) had stronger α-glucosidase inhibitory activity (IC_50_ value of 5.4 μM), but eurothiocins *F* (**36**) and eurothiocins G (**37**) displayed lower activities with IC_50_ values of 33.6 μM and 72.1 μM, respectively.

Alcantara et al. (2019) reported the anti-diabetic effects of various extracts of *Fuscoporia torulosa* MFSLP-12. A methanol extract of this fungus had significant α-glucosidase inhibitory activity, with an inhibition rate of 56%, and α-amylase inhibitory activity with inhibition rate of 38%. The IC_50_ value was about 5-fold and 9-fold higher for α-glucosidase and α-amylase than the control drug acarbose.

## Applications of microbial α-glucosidase and α-amylase inhibitors

### Diabetes

Type 2 diabetes mellitus is a genetically heterogeneous metabolic disorder characterized by high blood glucose levels, and insulin is the pancreatic hormone that controls blood sugar (DeFronzo, 2004; Artasensi et al., 2020). There is no cure for diabetes currently, but recent studies proves that diabetes morbidity and mortality can be controlled with optimal medical therapy, a healthy diet, and physical exercise (Asano, 2003).

#### Pharmacological therapy

Reducing postprandial hyperglycemia is the most important treatment for diabetes. The researchers realized that the inhibition of glucosidase with inhibitors could regulate the absorption of carbohydrates and prevent postprandial hyperglycemia (Asano, 2003). Those inhibitors could cause the release of GLP-1 and reduce glycated hemoglobin levels (Kumar and Sinha, 2012). Glucosidase inhibitors, such as acarbose, voglibose, and miglitol, are important first-line agents for type 2 diabetes patients (Hossain and Pervin, 2018; Dhatariya, 2019). Also, these drugs can be used as second-line agents in combination with metformin, which could reduce the dosage of metformin and improve safety (Clissold and Edwards, 1988; Chan et al., 2018).

**Figure 3:**
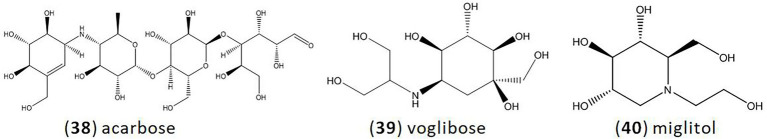
Clinical medicine of glycosidase inhibitors. (**38**).acarbose; (**39**).voglibose; (**40**).miglitol.

Acarbose (**38**), isolated from *Actinoplanes* sp. SE50, was the first commercialized glucosidase inhibitor launched in 1990 (Schmidt et al., 1977; Joshi et al., 2014). It is one of the most common glycosidase inhibitors, and also the most widely studied one. Acarbose inhibits many glycosidases, such as α-amylase, maltase, and glucoamylase, which could reduce the hydrolysis of starch in intestine (Liu and Ma, 2017; Li et al., 2022). Voglibose (**39**), isolated from *Streptomyces hygroscopicies* limonons, was discovered in 1981 (Japan), and marketed for clinical use since 1994 (Horii et al., 1986; Saito et al., 1998). It is a more tolerated and potent inhibitor of α-glycosidase than acarbose with fewer side effects and higher activities (Dabhi et al., 2013). Miglitol (**40**) was developed by Bayer and first marketed in 1998 (Kumar and Sinha, 2012). It is a derivative of 1-desoxynojirimycin (DNJ). DNJ can be isolated from *S. lavendulae* or other strains, and then chemically synthesized to form miglitol (Sels et al., 1999). Miglitol is completely absorbed by the small intestine with high bioavailability, whereas acarbose and voglibose are poorly absorbed with low bioavailability (Akmal and Wadhwa, 2022; Figure 3).

#### Dietary supplements

The presence of glycosidase inhibitors in the diet can inhibit the activity of human glycosidase and reduce the absorption of dietary carbohydrates (Park et al., 2021). In addition, there has been increasing concern about the possibility of using dietary supplements to prevent diabetes. For example, *Salacia reticulata* is used as a diabetic supplement in Japan (Asano, 2003; Zou et al., 2019).

### Obesity

Diet control is an important way to control obesity. Glycosidase are responsible for carbohydrate digestion, and increase postprandial blood sugar levels. Glycosidase inhibitors are potential compounds that can be used in weight loss. They inhibit glucosidase, delay the absorption of carbohydrates, and reduce people’s postprandial blood sugar levels and insulin responses to dietary carbohydrates (Peddio et al., 2022).

Also, another important factor for obesity is the abnormal differentiation or adipocytes dysfunction. Li et al. illustrated that DNJ, an α-glucosidase inhibitor, can inhibit adipogenesis during the differentiation of white preadipocytes, providing a new approach to explain the beneficial effects of α-glucosidase inhibitor on obesity (Li et al., 2019).

### Antiviral treatment

N-nonyl-deoxynojirimycin (NN-DNJ, α-glucosidase inhibitor derivative) is a potential antiviral drug. Block et al. reported that NN-DNJ induces misfolding of the hepatitis B virus envelope glycoproteins and further prevents virus formation (Block et al., 1998). Moreover, Zitzmann et al. demonstrated that NN-DNJ could prevent the formation and secretion of bovine viral diarrhea virus, a model for human hepatitis C virus (Zitzmann et al., 1999; Whitby et al., 2004). Also, N-butyl-deoxynojirimycin (α-glucosidase inhibitor) and its derivatives show significant antiviral against Ebola virus *in vitro*. It inhibits assembly and secretion of virus particle (Chang et al., 2013; Yuan, 2015).

### Lysosomal storage diseases

The lysosomal storage diseases are a group of inherited diseases that lead to metabolic disorders of the lysosomes. The diseases mainly include Fabry disease, Gaucher disease, Niemann-Pick disease and so on (Khanna et al., 2010; Mechtler et al., 2012). Giraldo et al. reported the treatment of type 1 Gaucher disease with N-butyl-deoxynojirimycin (α-glucosidase inhibitor) over 12 years. Eventually 80% of patients achieved the treatment goals, with stable levels of hematologic counts and volumes of the liver and spleen (Giraldo et al., 2018).

## Conclusion and further research

In recent years, the incidence of type 2 diabetes has been growing rapidly. Glucose, which is hydrolyzed by glycosidases, is absorbed into the blood, and then caused severe postprandial hyperglycemia. So glycosidase is an important therapeutic target for diabetes (Usman et al., 2019). However, marketed inhibitors of glycosidase have many side effects. Therefore, novel glycosidase inhibitors that are safer, more effective, and more cost-effective are needed.

Glycosidases are produced by microbes, animals and plants (Aslan et al., 2018; Demir et al., 2018). However, inhibitors of glycosidase are mainly derived from microbes. In the most recent 5 years, increasing research on new microorganisms producing inhibitors of glycosidase has been reported. Compared to general microorganisms, most new microorganisms are extremophiles, which is reflected by the number of papers on new microorganisms. This new focus on extremophiles expands the scope of the search glycosidase inhibitors. Five reports introduced nine new compounds with inhibitory activities against glycosidases in recent 5 years. They are compound **14** isolated from the marine fungus *Aspergillus terreus* OUCMDZ-2739 by Sun et al.; five new compounds **15–19** from a marine fungus *Penicillium* sp. by Guo et al.; novel xanthone (**21**) isolated from endophytic *Penicillium canescens* strain by Malik et al.; asperchalasine I (**30**) isolated from the mangrove fungus *Mycosphaerella* sp. by Qiu et al.; and Nigronapthaphenyl (**34**) extracted from the endophytic fungus *Nigrospora sphaerica* by Ukwatta et al. All of them come from marine and endophytic fungus. It would be very useful if a database for microorganisms and its glycosidase inhibitors was established.

In addition to applications in the treatment of diabetes and obesity, several applications for glycosidase inhibitors have been reported but have not yet been industrially developed. We look forward to seeing the use of these inhibitors expand.

## Author contributions

XW, JL, and JS reviewed conceptualization and wrote the manuscript. JB, KW, JL, and ZY collected the data from previous researches and prepared the figures and tables. HO designed of the work and reviewed critically for important intellectual content. LS designed and supervised the paper. All authors contributed to the article and approved the submitted version.

## Funding

This research was financially supported by the Natural Science Foundation of Shanghai (20ZR1424600), the National Natural Science Foundation of China (81773616), the Shanghai Excellent Technology Leader Program (17XD1423200), and Nature Science Foundation of Jiangsu Higher Education Institutions of China (20KJB180002).

## Conflict of interest

The authors declare that the research was conducted in the absence of any commercial or financial relationships that could be construed as a potential conflict of interest.

## Publisher’s note

All claims expressed in this article are solely those of the authors and do not necessarily represent those of their affiliated organizations, or those of the publisher, the editors and the reviewers. Any product that may be evaluated in this article, or claim that may be made by its manufacturer, is not guaranteed or endorsed by the publisher.
